# Distinct pathological phenotypes of Creutzfeldt-Jakob disease in recipients of prion-contaminated growth hormone

**DOI:** 10.1186/s40478-015-0214-2

**Published:** 2015-06-25

**Authors:** Ignazio Cali, Cathleen J. Miller, Joseph E. Parisi, Michael D. Geschwind, Pierluigi Gambetti, Lawrence B. Schonberger

**Affiliations:** Department of Pathology, Case Western Reserve University, School of Medicine, Cleveland, OH USA; Department of Clinical and Experimental Medicine, Second University of Naples, Naples, Italy; Department of Neurology, Kaiser Permanente Sunnyside Medical Center, Clackamas, OR USA; Departments of Laboratory Medicine & Pathology and Neurology, Mayo Clinic, Rochester, MN USA; Department of Neurology, Memory and Aging Center, University of California, San Francisco, CA USA; Division of High Consequence Pathogens and Pathology, National Center for Emerging and Zoonotic Infectious Diseases, Centers for Disease Control and Prevention, Atlanta, GA USA

**Keywords:** Iatrogenic Creutzfeldt-Jakob disease, PrP^Sc^, Growth hormone, Dura mater, Pathological phenotype

## Abstract

**Introduction:**

The present study compares the clinical, pathological and molecular features of a United States (US) case of growth hormone (GH)-associated Creutzfeldt-Jakob disease (GH-CJD) (index case) to those of two earlier referred US cases of GH-CJD and one case of dura mater (d)-associated CJD (dCJD). All iatrogenic CJD (iCJD) subjects were methionine (M) homozygous at codon 129 (129MM) of the prion protein (PrP) gene and had scrapie prion protein (PrP^Sc^) type 1 (iCJDMM1).

**Results:**

The index subject presented with ataxia, weight loss and changes in the sleep pattern about 38 years after the midpoint of GH treatment. Autopsy examination revealed a neuropathological phenotype reminiscent of both sCJDMV2-K (a sporadic CJD subtype in subjects methionine/valine heterozygous at codon 129 with PrP^Sc^ type 2 and the presence of kuru plaques) and variant CJD (vCJD). The two earlier cases of GH-CJDMM1 and the one of dCJDMM1 were associated with neuropathological phenotypes that differed from that of the index case mainly because they lacked PrP plaques. The phenotype of the earlier GH-CJDMM1 cases shared several, but not all, characteristics with sCJDMM1, whereas dCJDMM1 was phenotypically indistinguishable from sCJDMM1. Two distinct groups of dCJDMM1 have also been described in Japan based on clinical features, the presence or absence of PrP plaques and distinct PK-resistant PrP^Sc^ (resPrP^Sc^) electrophoretic mobilities. The resPrP^Sc^ electrophoretic mobility was, however, identical in our GH-CJDMM1 and dCJDMM1 cases, and matched that of sCJDMM1.

**Conclusions:**

Our study shows that receipt of prion-contaminated GH can lead to a prion disease with molecular features (129MM and PrP^Sc^ type 2) and phenotypic characteristics that differ from those of sporadic prion disease (sCJDMM1), a difference that may reflect adaptation of “heterologous” prion strains to the 129MM background.

## Introduction

The first case of iatrogenic Creutzfeldt-Jakob disease (iCJD) was described in 1974, in a recipient of corneal graft from a donor who died following a rapidly progressive dementing illness [1]. Subsequently, other mechanisms of iatrogenic transmission were identified worldwide. Contaminated neurosurgical instruments and electrodes used in stereotactic electroencephalography were shown to cause CJD in humans and experimentally in non-human primates [2–5]. More than 230 cases, mostly in France, of iCJD have been linked to injection of products extracted from cadaveric pituitary glands, including growth hormone (GH) (at least 226 cases) and gonadotropin (4 cases). Worldwide, a similar number of iCJD cases (at least 228 cases) have been associated with receipt of dura mater grafts, especially in Japan where greater than 60 % of all dura mater (d)-associated CJD (dCJD) cases have been identified [6, 7]. More recently, blood products from subjects who were later diagnosed with variant CJD (vCJD) have also been reported to transmit vCJD, resulting in iCJD cases [8–10]. Unlike the vast majority of vCJD cases, the mechanism of which is based on exogenous scrapie prion protein (PrP^Sc^) crossing the species barrier, iCJD is entirely a human disease which is transmitted from person to person.

The detailed pathology of GH-associated CJD (GH-CJD) with methionine (M) homozygous genotype at codon 129 (129MM) of the prion protein (PrP) gene has been reported in 1993–1994 in three French cases who had brain kuru plaques and detectable proteinase K (PK)-resistant PrP^Sc^ (resPrP^Sc^) [11] or positive PrP immunostaining [12]. Information on the type of resPrP^Sc^ (e.g., type 1 and type 2), however, was not available until 1994 [13]. More recently, a GH-CJDMM case with resPrP^Sc^ type 1 (GH-CJDMM1) was reported to show the diffuse or “synaptic” PrP immunostaining and no PrP plaques [14].

We have studied the clinical, pathological and molecular features of a United States (US) GH-CJD case that died in 2013 (index case) and compared the features of the index case to those of two earlier cases of GH-CJD and one dCJD case [15] referred to the US National Prion Disease Pathology Surveillance Center (NPDPSC) (Table 1). All four patients shared codon 129MM genotype and resPrP^Sc^ type 1. From this comparative study, two pathological phenotypes were observed in GH-CJDMM1; the first phenotype was characterized by different types of PrP aggregates, including kuru plaques, and some histopathological features of vCJD, such as florid plaques and “stellate” cells highlighted by the pericellular deposition of PrP [16]. This phenotype therefore shared features of both sCJDMV2-K and vCJD and was observed in our index case [17–19]. The second phenotype, observed in the two other GH-CJDMM1 cases, was characterized by the absence of PrP plaques and had several, but not all, characteristics of sCJDMM1. Finally, the dCJDMM1 case showed a phenotype indistinguishable from that of sCJDMM1 (Table 1).

**Table 1:** Clinical, molecular and pathological features of the iatrogenic CJD patients of this study and sporadic CJDMM1

Prion disease	GH-CJDMM1	GH-CJDMM1	GH-CJDMM1	dCJDMM1	sCJDMM1 (203 cases)^a^
Case	1 (index case)	2	3	4	
Clinical data					
Disease presentation	Ataxia, weight loss and insomnia.	Ataxia and slurred speech.	Left leg numbness with broad based gait.	Visual changes and ataxic gait.	Rapidly progressive cognitive decline; occasionally cortical visual disturbances, ataxia, and myoclonus.
Age (years)/gender	50/male	54/male	40/female	39/female	65.5^b^ (42–91)^c^
Disease duration (months)	14	2	2	4	3.9^b^ (1–18)^c^
Incubation time (years)	38^d^	41.5^d^	26.3^d^	6^e^	
Molecular features					
resPrP^Sc^ type	1	1	1	1	1
Codon 129 genotype	Met/Met	Met/Met	Met/Met	Met/Met	Met/Met
Histopathology (differences with sCJDMM1 underlined)					
PrP plaques					
Kuru	+++	-	-	-	-
Florid	+	-	-	-	-
Eosinophilic	+++	-	-	-	-
SD^f^/gliosis					
Severity					
Hippocampus	++/−	−/−	−/−	−/−	−/−
SN^g^ of midbrain	++/+++	−/−	−/−	−/−	−/−
Topography (SD only)	Severe in subcortical regions; preferentially in layers IV-VI of cerebral cortex (cc); cerebellar molecular layer, focal.	Less severe in occipital than frontal cc; entorhinal cc and basal ganglia spared; occasionally most severe in deep layers of the cc; cerebellar molecular layer, focal.	More severe in occipital than frontal cc; entorhinal cc and basal ganglia affected; occasionally most severe in deep layers of cc; cerebellar molecular layer, ubiquitous.	All layers of cc affected; more severe in occipital than frontal cc; entorhinal cc and basal ganglia affected; cerebellar molecular layer, focal.	All layers of cc affected; more severe in the occipital than frontal cc; entorhinal cc and basal ganglia affected; cerebellar molecular layer, focal.
PrP IHC^h^ cerebellum	PrP “stellate”, kuru plaques, plaque-like; occasionally “brush stroke-like”.	“brush stroke-like”	“brush stroke-like”	“brush stroke-like”	“brush stroke-like”

^a^[19, 46]; ^b^mean; ^c^range; considered from the ^d^the midpoint of treatment with GH or ^e^the time of grafting surgery to the appearance of the first clinical sign/symptoms; ^f^SD: spongiform degeneration; ^g^SN: substantia nigra; ^h^IHC: immunohistochemistry; (−) absent, (+) mild, (++) moderate, (+++) severe

Two different phenotypes have also been described in Japanese cases of dCJD, which were referred to, depending on the presence or absence of PrP plaques, as “plaque-type dCJD” (p-dCJD) or “non-plaque-type dCJD” (np-dCJD). Both groups shared the same codon 129MM genotype (one np-dCJD and one p-dCJD were 129MV) although the unglycosylated resPrP^Sc^ isoform in p-dCJD had about 1 kDa faster mobility than unglycosylated resPrP^Sc^ from np-dCJD and sCJDMM1 (e.g., ~20 kDa vs. ~21 kDa, respectively) [20]. The NPDPSC dCJDMM1 case had pathological and molecular features (electrophoretic mobility of resPrP^Sc^) that were indistinguishable from those of sCJDMM1, similar to the np-dCJD cases.

## Materials and methods

### Reagents and antibodies

Dulbecco’s Phosphate Buffered Saline (DPBS), Phenylmethanesulfonyl fluoride (PMSF) and Proteinase K were from Sigma-Aldrich (St. Louis, MO, USA). Tween 20, 10X Tris/glycine, 10X Tris/Glycine/SDS, 2X Laemmli Sample Buffer, β –mercaptoethanol and Criterion 15 % Tris–HCl polyacrylamide precast gels were from Bio-Rad Laboratories (Hercules, CA, USA). Odyssey blocking buffer, Infrared Dye (IRDye) 800CW goat anti-mouse IgG (1 mg/ml), IRDye 680RD goat anti-rabbit IgG (1 mg/ml) and the Polyvinylidene difluoride (PVDF) membrane (Immobilon-FL) were from LI-COR Biosciences (Lincoln, NE, USA). The 3F4 monoclonal antibody to PrP residues 106–110 [21, 22], 12B2 monoclonal antibody to PrP residues 89–93 (this epitope is not preserved in resPrP^Sc^ type 2) [23], and the polyclonal antibody Tohoku-2 to PrP residues 97–103 [24] were used in this study.

### Tissue samples and subject selection

At brain autopsies, one half was frozen and stored at −80 °C and the other half was fixed in formalin for neuropathological examination. Brain tissue was sent to the NPDPSC for analysis. Frozen brain tissue used for the molecular study was from the frontal cortex [GH-CJDMM1 (N = 3), dCJDMM1 (N = 1), sCJDMM1 (N = 3), sCJDMV2-K (N = 3)] and cerebellum [GH-CJDMM1 (N = 2), dCJDMM1 (N = 1), sCJDMM1 (N = 3), sCJDMV2-K (N = 3)]. Subjects were diagnosed as sCJDMM1 and sCJDMV2-K according to Parchi et al. [19, 25]. Mean ages at disease onset and disease durations were 70 ± 15 (standard deviation, SD) years and 2 ± 0.1 (SD) months in sCJDMM1, and 64 ± 9 (SD) years and 10 ± 3 (SD) months in sCJDMV2-K, respectively. These two sCJD subtypes (sCJDMM1 and sCJDMV2-K) were chosen as control cases for two reasons: (1) all iatrogenic CJD (iCJD) cases in this study shared with sCJDMM1 the 129 genotype and resPrP^Sc^ type 1; (2) one of the three iCJD cases (the index case, case 1 of Table 1) shared some histological and immunohistochemical features with sCJDMV2-K. In addition to case 1, the GH-CJDMM1 (cases 2 and 3, Table 1) and dCJD subjects (case 4, Table 1) [15] reviewed at the NPDPSC had age at disease onset of 54, 40 and 39 years, disease duration of 2, 2 and 3 months and incubation period (from mid-point of GH-therapy or receipt of the dural graft) of 41.5, 26.3 and 6 years, respectively. All patient protocols were approved by the Institutional Review Boards of the University Hospitals Case Medical Center.

### Preparation of brain tissues and proteinase K digestion

A 20 % (wt/vol) brain homogenate (BH) in 1X Dulbecco’s PBS (DPBS) was mixed 1:1 with the 2X LB100 buffer (1X LB100: 100 mM NaCl, 0.5 % Nonidet P-40, 0.5 % sodium deoxycholate, 10 mM EDTA, 100 mM Tris–HCl, pH 8.0) [26, 27]. The resulting 10 % BH was centrifuged at 1000 g for 5 minutes and the supernatant (S1) was collected. The S1 was incubated with proteinase K (PK) at 37 °C for 1 hour. The PK concentration used was equal to 10 U/ml [48 U/mg specific activity at 37 °C, with 1 U/ml equal to 20.8 mg/ml PK]. The enzymatic reaction was stopped with 3 mM PMSF. Each sample was mixed 1:1 with the 2X Laemmli sample buffer (6 % SDS, 20 % glycerol, 4 mM EDTA, 5 % β –mercaptoethanol, 125 mM Tris–HCl, pH 6.8) and denatured for 10 minutes at 100 °C.

### Western blot analysis

Proteins were loaded onto a 15 % Criterion Tris–HCl polyacrylamide precast gel for gel electrophoresis, blotted into the Immobilon-FL PVDF membranes for 2 hours, blocked with the Odyssey Blocking Buffer for 45 minutes and incubated with the 3F4 (1:20,000), 12B2 (0.1 μg/ml) or Tohoku 2 (1:20,000) for 2 hours. Membranes were washed with DPBS-T (1X DPBS with 0.1 % Tween 20) and incubated with IRDye 800CW goat anti-mouse IgG (1:15,000) or IRDye 680RD goat anti-rabbit IgG (1:15,000) for 1 hour. After washing with DPBS-T, membranes were developed by Odyssey infrared imaging system (LI-COR Biosciences).

### Histology and PrP immunohistochemistry

Histopathology and PrP immunohistochemistry were performed as previously described [28]. Briefly, deparaffinized and rehydrated sections were immersed in Tris-buffered saline-Tween 20 (TBS-T) and endogenous peroxidase blocked by Envision Flex Peroxidase Blocking Reagent (Dako) for 10 minutes. Sections were washed, immersed in 1.5 mmol/L hydrochloric acid, microwaved for 15 minutes and incubated with 3F4 (1:1000) for 1 hour. After washing and incubation with Envision Flex/HRP polymer for 30 minutes (Dako), sections were treated with Envision Flex DAB (Dako) to show the immunostaining.

### Genetic analysis

DNA was extracted from frozen brain tissues and PrP gene analysis performed as previously described [18, 29].

## Results

### Clinical history of the index case

**Fig. 1:**
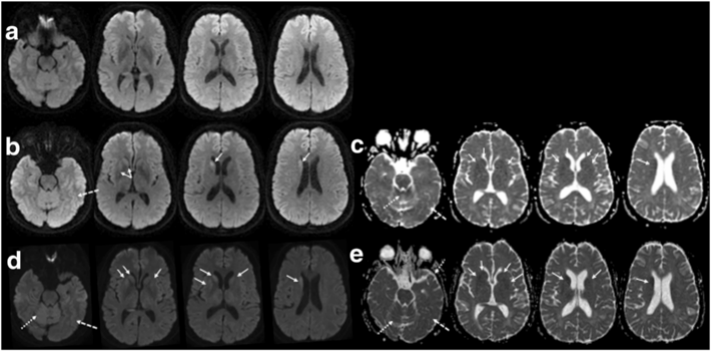
Brain MRIs of index case. **a**-**e**: MRI was normal at 4 months without clear evidence of restricted diffusion on DWI sequence (**a**), but progressed to restricted diffusion in the right caudate head (solid arrows) and more subtle cortical (dashed arrows) and possible superior vermis (dotted arrows) involvement at 10 months (**b**, DWI; **c**, ADC map), with progression to bilateral asymmetric caudate and more evident cerebellar vermis involvement at 11.5 months (**d**, DWI; **e**, ADC map)

A 50-year-old athletic male presented to his primary physician with 18 months of erectile dysfunction, three months of chronic dry cough, two months of right toe numbness, unintentional 14 pounds weight loss over the prior eight months, and one month (“onset”) of leg fatigue and instability, disequilibrium (“like on a boat”), and insomnia (early awakening). Running and jumping had become difficult, and he had fallen during sports. Physical examination reportedly was normal. Past medical history was notable for idiopathic isolated growth hormone deficiency treated with human growth hormone from 6.5 to 18 years of age (received pituitary-derived human growth hormone from the US National Hormone and Pituitary Program between 1969 and 1980). At six weeks after motor onset he developed frequent hot flashes, with whole-body sweating. At three and a half months, at his first neurology consultation, he could no longer run and had trouble using stairs due to imbalance, was having intermittent episodes of horizontal eye jerking with downgaze lasting seconds, intermittent difficulty concentrating and remembering new names, mild intermittent dysphagia, and a few episodes of leg jerking mostly with startle, but his weight loss had stabilized. His detailed neurological exam was normal except for finding tandem gait uncharacteristically challenging, moderate sway on Romberg, and decreased cold perception below the ankles. By 5 months, he had worsening of gait instability, insomnia, myoclonus/startle, and numbness/tingling (spread to bilateral toes and soles), and he noted muscle spasms/cramps as well as exercise-induced body tremulousness. Exam showed slight head tremor with visual pursuit, mild arm paratonia, spastic/ataxic gait, inability to tandem, positive Romberg. By 6 months, he developed constipation, urinary retention, worsening of “bouncing eyes,” intermittent head jerking back, and exam showed new pendular nystagmus with vertical gaze, asymmetric upper extremity postural and intention tremor, brisk patellar reflexes, absent Achilles reflexes, diminished distal vibration and pin prick perception, and slight orthostasis by pulse. By 7 months, he required catheterization for neurogenic bladder. Paresthesias had ascended to the sacral level. By 9 months, he was anxious with pressured speech, walker dependent with orthostatic lower extremity tremor and appendicular ataxia. He was wheelchair bound by 10 months with hypophonia and intermittent focal and generalized myoclonus. By 11 months, he had short term memory loss, daytime hypersomnolence, polymyoclonus and nightmares disrupting sleep, worsening dysphagia and dysautonomia with anejaculation, and he had to stop working primarily because of “bouncing eyes” affecting vision. Extensive evaluation for autoimmune, infectious, toxic, metabolic, neoplastic, paraneoplastic/antibody-mediated, and genetic (spinocerebellar ataxias 1,2,3, and 6) causes of his symptoms was unremarkable except for a transient elevation in venous lactate, mildly elevated aldolase of 8.4 (normal < =8.1 unit/L), hemoglobin A1c 5.9-6.0 (5.7-6.4 prediabetes range), high B-6 of 49.8 ng/mL (normal 2.1-21.7 ng/mL), low thiamine of 6 (normal 8–30 nmol/L), positive serum striational antibody 1:160, felt to be false-positive [30]. Cerebrospinal fluid (CSF) at six months showed normal cells, protein, glucose, high intermediate neuron-specific enolase of 30 ng/ml (15–30 ng/ml intermediate, > 30 consistent with CJD, Mayo Laboratories, Rochester, MN) and 14-3-3 negative (<1.0 ng/mL, Mayo). CSF 14-3-3 was positive (Western Blot) and total-tau elevated to 1517 pg/ml; (>1150 is consistent with CJD per NPDPSC) at 11.5 months. Brain magnetic resonance imaging (MRI) was normal at 4 months, progressed to restricted diffusion in the right caudate head and more subtle cortical and possible superior vermis involvement at 10 months, with progression to bilateral asymmetric caudate and clear cerebellar vermis involvement at 11.5 months (Fig. 1). Notably, the formal reports missed the cerebellar and cortical involvement. Electroencephalogram (EEG) at 11 months was normal except for severe polymyoclonus causing arousals from stage 2 sleep; at 11.5 months it showed occasional, very mild irregular slowing of the background (6–8 Hz) during wakefulness without periodic discharges. EMG/NCS at six months showed a non length-dependent sensorimotor polyneuropathy, and essentially unchanged at 8 months. Vastus lateralis muscle biopsy (10 months) showed minimal recent neurogenic muscle atrophy without evidence of mitochondrial or other primary myopathy. His cognitive impairment, daytime hypersomnolence, myoclonus, subjective “eye jiggling,” anejaculation worsened severely and he died 14 months after motor onset. Brain-only autopsy was performed two days post-mortem.

### Histopathology

#### Index case

**Fig. 2:**
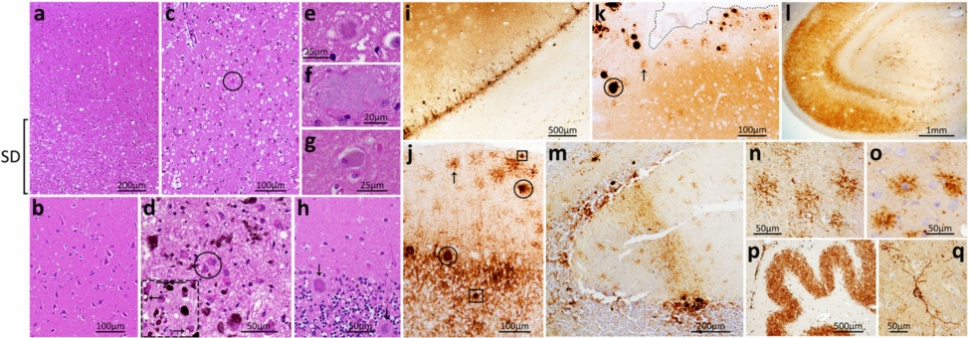
Histopathology and immunohistochemistry of the index US GH-CJD patient. **a**-**h**: Histopathology. **a**-**c**: Spongiform degeneration (SD) selectively affecting the deeper layers of the cingulate gyrus (**a**; bracket), the hippocampal gyrus (**b**) and more severely the basal ganglia (**c**). **c**-**d**: Reactive astrogliosis (circles) in basal ganglia (**c**) and midbrain substantia nigra (**d**); arrows in the inset (dashed square) indicate intraneuronal vacuole formation. **e**: A kuru plaque in the frontal cortex reminiscent of the florid plaques observed in vCJD. **f**: A different type of plaque, apparently core-free, was detected in the cortical (**f,** parietal cortex) and subcortical brain regions. (not shown) **g**: A ballooned neuron. **h**: Kuru plaques (arrows) in the cerebellar granular layer. **i**-**q**: Immunohistochemistry. **i**: Laminar PrP immunostaining of the insula. **j**: Kuru plaque (circles) and plaque-like (squares) PrP immunostaining in the cerebellar granular and molecular layers, and peri-cellular PrP (“stellate”) (arrow) in the molecular layer. **k**: Large PrP aggregates (circle) and peri-cellular PrP immunostaining (“stellate” cells) (arrow) in proximity of one sulcus in the frontal cortex (dotted line delimiting the sulcus). **i**: Intense PrP immunostaining of the hippocampus. **m**: “Brush stroke-like” PrP immunostaining in the molecular layer of the cerebellum. **n**-**o**: Higher magnification of the PrP-immunostained “stellate” cells in the cerebellar molecular layer of the present case (**n**) and in one vCJD case (**o**). **p**-**q**: Intense PrP staining of the dentate nucleus (**p**) and along processes around the perikaryon of a neuron of the frontal cortex (**q**). Scale bar of inset in **d**: 50 μm; antibody: 3F4

Gliosis and spongiform degeneration (SD) affected the gray matter of the entire brain although the basal ganglia, thalamus and midbrain showed more severe pathological changes than the cerebral cortex where SD had a laminar distribution affecting preferentially layers IV-VI (Fig. 2a-d; case 1, Table 1). Furthermore, “ballooned” neurons were occasionally observed in the cerebral cortex (Fig. 2g). The cerebellum showed focal atrophy with severe astrogliosis and loss of granule cells. Three types of abnormal PrP aggregates were observed: i) kuru plaques, occasionally in clusters, in the granular layer and, less commonly, in the cerebral cortex and molecular layer and deep white matter of the cerebellum; ii) kuru plaques surrounded by vacuoles, resembling the florid plaques of variant CJD (vCJD), in the cerebral cortex; iii) eosinophilic plaque-like formations, often populated the superficial cortical layers, were generally larger than, and lacked a dense core and the pale peripheral halo found in, kuru plaques. (Fig. 2e-f, h).

Immunohistochemical examination revealed that the PrP deposition was widespread throughout the brain; in the cerebral cortex, it co-distributed with the laminar SD and was either diffuse or distributed in plaque-like and perineuronal patterns (Fig. 2i). The large and eosinophilic plaque-like formations observed by hematoxylin and eosin (HE) examination were intensely immunostained (Fig. 2k). In the cerebellum, kuru plaques and plaque-like formations also were very immunoreactive (Fig. 2j). Finally, the cerebellar molecular layer as well as cortical and subcortical brain regions showed an unusual pericellular PrP deposition that highlighted cells apparently characterized by a round perikaryon surrounded by short branching processes (Fig. 2j-k, n-o). Similar cells have been observed in variant CJD (vCJD) [16]. Occasionally, the cerebellar “brush stroke-like” pattern of PrP deposition typical of the sCJDMM1 subtype was also observed (Fig. 2m).

### The three earlier US iatrogenic CJD (iCJD) patients

**Fig. 3:**
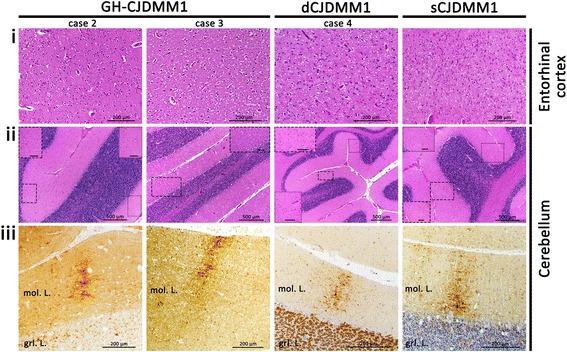
Histopathology and immunohistochemistry of GH-CJDMM1 (cases 2 and 3), dCJDMM1 (case 4) and sCJDMM1. Histopathology (rows **i**-**ii**) and PrP immunohistochemistry (row **iii**). **i**: No spongiform degeneration (SD) (case 2) and fine SD (cases 3, 4 and sCJDMM1). **ii**: Focal SD (cases 2, 4 and sCJDMM1) and ubiquitous SD (case 3). Insets contain magnified regions delimited by rectangles in the main figure. **iii**: “Brush stroke-like” PrP immunostaining pattern (cases 2, 3, 4 and sCJDMM1). Scale bar of insets in **ii**: 100 μm; mol. L.: molecular layer; grl. L.: granular layer; antibody in **iii**: 3F4

The pathological examination of two GH-CJDMM1 patients (cases 2 and 3, Table 1) revealed fine SD affecting occasionally the deeper layers of the cerebral cortex; the hippocampus was spared in both cases whereas the entorhinal cortex and anterior basal ganglia were virtually spared and the occipital cortex had less severe SD than frontal cortex in case 2. In the cerebellum, SD was focal in case 2 and ubiquitous in case 3 (Fig. 3, rows i-ii and data not shown). In dCJDMM1 (case 4, Table 1), a fine SD affected homogeneously all layers of the cerebral cortex with sparing of the hippocampus and focal distribution in the cerebellum. All three iCJD cases were free of plaques (Fig. 3, rows i-ii). Immunohistochemical examination revealed the presence synaptic PrP deposition in the brain, which showed the “brush stroke-like” PrP pattern in the cerebellar molecular layer of cases 2, 3 and 4 (Fig. 3, row iii).

### PrP^Sc^ type determination

#### Index case

**Fig. 4:**
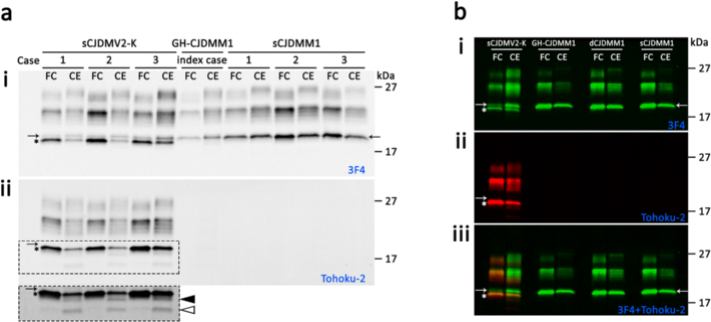
Western blot of the PK-resistant PrP^Sc^. Brain homogenates from the frontal cortex (FC) and the cerebellum (CE) of (**a**) the index case, (**b**) GH-CJDMM1 (case 2), dCJDMM1 (case 4) and (**a** and **b**) control cases sCJDMV2-K and sCJDMM1 were treated with 10 U/ml PK (~200 μg/ml) prior immunoblotting with antibodies (**i**) 3 F4, (**ii**) Tohoku-2, or (**iii**) 3 F4- and Tohoku-2-merged immunoreactivities. **i**: The unglycosylated resPrP^Sc^ from the index case co-migrated at ~20 kDa with the corresponding resPrP^Sc^ type 1 of sCJDMM1 and with a ~20 kDa fragment of sCJDMV2-K (arrow). The asterisk in **i**-**iii** indicates the unglycosylated resPrP^Sc^ type 2. **ii**: Only the resPrP^Sc^ associated with sCJDMV2-K (asterisk) reacted with Tohoku-2 confirming that it belongs to type 2. Tohoku-2 immunoreacted also with a resPrP^Sc^ fragment of ~18.0 kDa and to a lower size fragment (indicated in **a** by the filled and empty arrowheads, respectively) in the cerebellum of sCJDMV2-K, but not with the resPrP^Sc^ ~20 kDa fragment. The dashed rectangle in **ii**-(**a**) is shown below at a longer exposure time. **iii**: Merged 3 F4 (green dye) and Tohoku-2 (red dye) immunoreactivities

The electrophoretic profile of the PK-resistant PrP^Sc^ (resPrP^Sc^) from this GH-CJDMM1 patient was examined in parallel with those of resPrP^Sc^ from sCJDMM1 (N = 3) and sCJDMV2-K (N = 3) subtypes with which the index cases shared the molecular features (sCJDMM1) and some aspects of the histopathological phenotype (sCJDMV2-K). The unglycosylated resPrP^Sc^ isoform of GH-CJDMM1 co-migrated at ~20 kDa with the unglycosylated resPrP^Sc^ type 1 associated with sCJDMM1. Probing with the antibodies Tohoku-2 and 12B2 to PrP^Sc^ type 2 and type 1, respectively, confirmed that the ~20 kDa contained exclusively PrP^Sc^ type 1. In contrast, two bands populated by unglycosylated resPrP^Sc^ fragments were observed in sCJDMV2-K: a prominent ~19 kDa band that matched in electrophoretic mobility resPrP^Sc^ type 2, and a ~20 kDa thinner band (Fig. 4a). Tohoku-2 confirmed the presence of resPrP^Sc^ type 2 in the ~19 kDa band but not in the ~20 kDa band of sCJDMV2-K. The ~20 kDa band has previously been described in subjects with the sCJDMV2-K subtype [25, 31].

#### Other iCJD patients

The unglycosylated resPrP^Sc^ from GH-CJDMM1 (cases 2 and 3, Table 1) and dCJDMM1 (case 4, Table 1) co-migrated at ~20 kDa with the unglycosylated resPrP^Sc^ type 1 of sCJDMM1 as well as the ~20 kDa fragment of sCJDMV2-K. The resPrP^Sc^ ~20 kDa fragments from all iCJD cases immunoreacted with 12B2 but not with Tohoku-2 antibody (Fig. 4b and data not shown).

## Discussion

Our index case presented with dysautonomia, a dry cough, neuropathy and cerebellar ataxia at age 50, approximately 38 years after growth hormone (GH) midpoint of treatment, and died 14 months after motor onset. Histopathological features were defined by the presence of 1) homogeneous eosinophilic plaques lacking a core, 2) typical kuru plaques occasionally surrounded by vacuoles (florid plaques), and 3) “stellate” cells highlighted by the pericellular deposition of prion protein (PrP). Spongiform degeneration (SD) with laminar distribution in the cerebral cortex was associated with plaque, plaque-like and perineuronal PrP immunostaining. PrP staining was also intense in the hippocampus, subcortical brain regions and dentate nucleus. Therefore, the neuropathological features of this case mimicked those of sCJDMV2-K in terms of the general topography and type of SD and PrP immunoreactivity, as well as the presence of kuru plaques. The neuropathological features differed from those of sCJDMV2-K, however, by the presence of the homogeneous “stellate” cells and occasionally florid plaques in the cortical regions and “brush stroke-like” staining in the cerebellum, the latter being a feature of sCJDMM1. Furthermore, this patient was homozygous for methionine at codon 129 (129MM) and showed the PK-resistant PrP^Sc^ (resPrP^Sc^) type 1, which in sCJD has very different pathological features [19].

Two previous GH-CJDMM1 cases that were reviewed at the NPDPSC presented at the age of 54 years with “progressive weakness, ataxia and slurred speech” (case 2) and at the age of 40 years with “progressive body stiffness and writhing that developed into left leg numbness and broad based gait” (case 3), approximately 41.5 years (case 2) and 26.3 years (case 3) after GH midpoint of treatment. Both these patients died after a 2 month disease course. Pathologically, both showed a phenotype reminiscent of sCJDMM1. Similar to sCJDMM1, both cases i) lacked amyloid plaques, ii) showed fine SD and “synaptic” PrP immunostaining that iii) spared the hippocampus. Unlike sCJDMM1, however, GH-CJDMM1 case 2 showed more severe SD in the frontal cortex rather than occipital cortex and sparing of the entorhinal cortex and anterior basal ganglia, whereas both cases (2 and 3) showed occasional laminar SD [26, 28].

A search of the literature yielded four GH-CJDMM cases with extensive pathological assessment, which at histopathological examination revealed either the presence (N = 3) [11, 12] or the absence (N = 1) [14, 32] of kuru plaques, and general histopathological phenotypes similar to those in our cases. Unfortunately, although the presence of resPrP^Sc^ was confirmed by western blot in three cases, typing of resPrP^Sc^ was not available in the GH-CJDMM cases with kuru plaques [11], whereas resPrP^Sc^ type 1 was demonstrated in GH-CJDMM lacking kuru or other types of plaques (GH-CJDMM1) [14, 32]. A positive PrP immunostaining was shown in the case report of Delisle et al. [12]. All four cases presented with ataxia but those with kuru plaques had ~ 6, 2.7 and 1.7 times longer disease duration than the GH-CJDMM1 without plaques (36, 16 and 10 months vs. 6 months). A longer disease duration also was observed in our GH-CJDMM1 index case (14 months). The two phenotypes associated with GH-CJDMM1 seem to share major pathological and clinical features with those reported in the Japanese dCJD cases. With the exception of two cases, all the Japanese dCJD share the 129MM genotype [33]. The plaque-associated cases (p-dCJD) are characterized clinically by ataxia and slow disease progression and pathologically by widespread PrP plaque deposition. The pathological phenotype of p-dCJD would therefore share common features with our index case with the exception of the “stellate” PrP staining that, to our knowledge, has not been reported in dCJD. Adding complexity to the plaque-associated phenotype, however, is that different combinations of PrP plaques (e.g., kuru, florid and eosinophilic) were observed in both p-dCJD [34–36] and in GH-CJDMM cases with plaques [11, 12]. The presence of florid plaques and “stellate” cells deposition in both GH-CJDMM1 and vCJD is intriguing. Florid plaques, the pathological hallmark of variant CJD (vCJD), also have been described in p-dCJD [20, 35, 37–40].

In contrast to the p-dCJD, the non-plaque-type dCJD (np-dCJD) cases were less likely to present clinically with ataxia and disease progression was rapid [7, 20]. In addition, the histopathological phenotype of the np-dCJD cases was indistinguishable from that of sCJDMM1; the np-dCJD phenotype thus would not fully match that of our GH-CJDMM1 without plaques. Also, as our dCJDMM1 and sCJDMM1 are indistinguishable, our dCJDMM1 and the Japanese np-dCJD would share the same histopathological phenotype.

A seemingly major discrepancy between our GH-CJDMM1 and the Japanese dCJDMM1 patients is represented by the size of the resPrP^Sc^. The ~20 kDa molecular size of the unglycosylated resPrP^Sc^ in our three GH-CJDMM1 cases matched that of the unglycosylated resPrP^Sc^ type 1 of sCJDMM1 (Fig. 4a-b) [26]. In contrast, the unglycosylated resPrP^Sc^ in Japanese dCJD migrated ~1 kDa faster in p-dCJD than np-dCJD (e.g., ~20 kDa vs. ~21 kDa, respectively) [33]. Moreover, resPrP^Sc^ from np-dCJD and Japanese sCJDMM1 was shown to co-migrate to ~21 kDa [33] although divergent data also have been reported [34, 35, 38, 40–42]. The ~1 kDa difference in gel mobility of resPrP^Sc^ type 1 of sCJDMM1 observed in our and Kobayashi’s experiments [33] may reflect differences in experimental conditions, such as the buffer pH and buffer capacity [27, 28]. It has been shown that PK digestion of PrP^Sc^ carried out at buffer pHs either < or > than 7.2 has a major effect on the mobility of resPrP^Sc^. At pHs < 7.2, the unglyc. resPrP^Sc^ type 1 from sCJDMM1 migrates to ~21 kDa whereas at pHs > 7.2 (our buffer pH is 8.0) resPrP^Sc^ type 1 migrates to ~20 kDa [26–28]. Thus, gel mobility of resPrP^Sc^ type 1 in sCJDMM1 is influenced by the pH.

It has been hypothesized that the phenotype with kuru plaques in p-dCJD is caused by a cross-sequence transmission of sCJDMV2 or sCJDVV2 prions to dura mater recipients with 129MM genotype, whereas the lack of kuru plaques in np-dCJD would be explained by an infection with prions from sCJDMM1 [33, 43, 44]. Because Japanese dCJD and our GH-CJDMM1 show similar major divergent pathological phenotypes, it is conceivable that a similar phenomenon occurred in GH-CJD, with pools of growth hormone being contaminated with different prion strains of sCJD. Of note, iCJD cases with sCJDVV1- or sCJDMM2-like phenotypes have not been reported, likely due to the rarity and poor transmissibility to humanized transgenic (Tg) mice of these two sCJD subtypes [45].

There are also several unusual clinical features of our index case that, to our knowledge, have not previously been reported in GH-CJD. These include MRI evidence of deep nuclei to cortical spread and his symptoms of dysautonomia, dry cough, and sensorimotor neuropathy. Interestingly, his dysautonomia began approximately 1.5 years prior to cerebellar symptoms. It is possible that these symptoms occurred in prior cases, but might not have been considered as part of the iCJD syndrome. When treatments become available, knowing the prodromal symptoms might allow earlier diagnosis and intervention.

## Conclusions

The similarity of the two phenotypes we observed in GH-CJDMM1 compared to the two phenotypes reported in dCJDMM1 suggests that despite the difference of the portal of entry and routes of propagation, the exogenous PrP^Sc^ triggering the disease undergoes a similar process of adaptation. Furthermore, it is possible that the exogenous PrP^Sc^ belonged to more than one strain of sCJD or the strain adaptation resulted in a hybrid strain. A detailed analysis of the molecular features in both groups of GH-CJDMM1 and a transmission study to humanized Tg mice would help to understand the molecular mechanism(s) underlying the two pathological phenotypes. Our study broadens the spectrum of possible pathological phenotypes among the GH-CJDMM1 patients and underscores the importance of a neuropathology surveillance system in obtaining new information and insights about the prion diseases.

## Abbreviations

NPDPSC: National Prion Disease Pathology Surveillance Center
CJD: Creutzfeldt-Jakob disease
iCJD: Iatrogenic CJD
vCJD: Variant CJD
sCJD: Sporadic CJD
GH-CJD: Growth hormone-associated CJD
dCJD: Dura mater-associated CJD
p-dCJD: Plaque-type dCJD
np-dCJD: Non-plaque-type dCJD
PrP: Prion protein
PrP^Sc^: Scrapie prion protein
resPrP^Sc^: Proteinase K (PK)-resistant PrP^Sc^
129MM: Methionine (M) homozygous at codon 129 of PrP gene
